# Persistent Effects of Developmental Exposure to 17α-Ethinylestradiol on the Zebrafish (*Danio rerio*) Brain Transcriptome and Behavior

**DOI:** 10.3389/fnbeh.2017.00069

**Published:** 2017-04-20

**Authors:** Tove Porseryd, Kristina Volkova, Nasim Reyhanian Caspillo, Thomas Källman, Patrik Dinnetz, Inger Porsh Hällström

**Affiliations:** ^1^School of Natural Sciences, Technology and Environmental Studies, Södertörn UniversityHuddinge, Sweden; ^2^Örebro Life Science Center, School of Science and Technology, Örebro UniversityÖrebro, Sweden; ^3^National Bioinformatics Infrastructure Sweden, Uppsala UniversityUppsala, Sweden; ^4^Science for Life Laboratory and Department of Medical Biochemistry and Microbiology, Uppsala UniversityUppsala, Sweden

**Keywords:** stress behavior, brain transcriptome, 17α-ethinylestradiol, RNA sequencing, developmental exposure, zebrafish, endocrine disrupting compounds, persistent effects

## Abstract

The synthetic estrogen 17α-ethinylestradiol (EE_2_) is an endocrine disrupting compound of concern due to its persistence and widespread presence in the aquatic environment. Effects of developmental exposure to low concentrations of EE_2_ in fish on reproduction and behavior not only persisted to adulthood, but have also been observed to be transmitted to several generations of unexposed progeny. To investigate the possible biological mechanisms of the persistent anxiogenic phenotype, we exposed zebrafish embryos for 80 days post fertilization to 0, 3, and 10 ng/L EE_2_ (measured concentrations 2.14 and 7.34 ng/L). After discontinued exposure, the animals were allowed to recover for 120 days in clean water. Adult males and females were later tested for changes in stress response and shoal cohesion, and whole-brain gene expression was analyzed with RNA sequencing. The results show increased anxiety in the novel tank and scototaxis tests, and increased shoal cohesion in fish exposed during development to EE_2_. RNA sequencing revealed 34 coding genes differentially expressed in male brains and 62 in female brains as a result of EE_2_ exposure. Several differences were observed between males and females in differential gene expression, with only one gene, *sv2b*, coding for a synaptic vesicle protein, that was affected by EE_2_ in both sexes. Functional analyses showed that in female brains, EE_2_ had significant effects on pathways connected to the circadian rhythm, cytoskeleton and motor proteins and synaptic proteins. A large number of non-coding sequences including 19 novel miRNAs were also differentially expressed in the female brain. The largest treatment effect in male brains was observed in pathways related to cholesterol biosynthesis and synaptic proteins. Circadian rhythm and cholesterol biosynthesis, previously implicated in anxiety behavior, might represent possible candidate pathways connecting the transcriptome changes to the alterations to behavior. Further the observed alteration in expression of genes involved in synaptogenesis and synaptic function may be important for the developmental modulations resulting in an anxiety phenotype. This study represents an initial survey of the fish brain transcriptome by RNA sequencing after long-term recovery from developmental exposure to an estrogenic compound.

## Introduction

17α-ethinylestradiol (EE_2_) is the most potent endocrine disrupting compound (EDC) pollutant contaminating the aquatic environment (Aris et al., 2014; Laurenson et al., 2014). Being 10-fold more active than estradiol itself (Thorpe et al., 2003; Denny et al., 2005), and having a predicted no adverse effect level of 0.1 ng/L for water-living organisms (Caldwell et al., 2012), its ubiquitous worldwide presence in sewage treatment plant effluents and reclaimed water is a matter of great concern (Mompelat et al., 2009; Aris et al., 2014). It is detected in effluents in levels from <1 ng/L (the present detection limit of such analyses) to as much as 2–300 ng/L (Ternes, 1998; Kolpin et al., 2002; Laurenson et al., 2014). EE_2_ is persistent in the environment and signs of bio-magnification have been observed (Aris et al., 2014).

In addition to the effects of EDCs on the reproductive axis in fish we and others have reported a significant influence also on non-reproductive behaviors in adult fish of importance for fitness, such as risky behavior, aggression, anxiety, and shoaling (Espmark Wibe et al., 2002; Majewski et al., 2002; Bell, 2004; Colman et al., 2009; Xia et al., 2010; Hallgren et al., 2011; Reyhanian et al., 2011; Dzieweczynski et al., 2014; Heintz et al., 2015). In recent studies, we found that low-dose EE_2_ exposure of both zebrafish and guppies during development resulted in increased anxiety as adults (Volkova et al., 2012, 2015b), suggesting irreversible changes of the neuroendocrine system. We have also found that the effects on anxiety behavior from developmental exposure of EE_2_ was transmitted to the first and the second generation of unexposed progeny in both guppy and zebrafish (Volkova et al., 2015a,b). Effects of EDCs at the level of tissue organization in the developing brain has in mammals been observed to affect fertility and behavior (McLachlan, 2001; Crews and McLachlan, 2006; Dickerson and Gore, 2007). Altered non-reproductive behavior in rodents developmentally exposed to EDC's have been shown to be transmitted over generations of unexposed progeny accompanied by alterations in the brain transcriptome (Skinner et al., 2008; Wolstenholme et al., 2012). Alterations in the epigenome, shown to be target of hormone modulations (Simerly et al., 1997; McCarthy et al., 2009; Casati et al., 2015; McCarthy and Nugent, 2015), are indicated as mediators of the changes in brain imprinting. Epigenetic mechanisms are dynamic and influenced by the environment, especially during the early life stages of an organism (Casati et al., 2015).

In this study, we analyze if persistent effects on gene expression in the zebrafish brain could be discerned in the adult fish after developmental EE_2_ exposure, accompanying an anxiogenic phenotype. We postulated that effects of anxiety should affect the regulation of genes related to the stress axis in the brain, but presumably also several other functions. To achieve this, we repeated the study mentioned above (Volkova et al., 2015b) with slightly different exposure protocol and, as it turned out, higher actual exposure levels. We analyzed three non-reproductive behaviors after 120 days remediation, and performed RNA sequencing of the complete brain transcriptome in male and female zebrafish. The purpose was to extend the understanding of the persistent effects of estrogenic substances that linger after discontinued exposure.

## Methods

### Animals and treatments

Fertilized zebrafish eggs from eight separate parental pairs of the wild type strain AB were obtained from the Karolinska Institute Zebrafish Core Facility, Solna, Sweden. Eggs from each parental pair were divided into three lots and assigned to treatment groups of 0, 3, and 10 ng EE_2_/L, respectively. EE_2_ (17α-ethinylestradiol, Sigma-Aldrich) was dissolved in acetone and stock solutions were mixed with pre-heated fish maintenance system water to final nominal concentrations of 0, 3, and 10 ng EE_2_/L. The nominal concentrations were considered relevant as they are within the range of concentrations measured in effluents and surface waters (Ternes, 1998; Kolpin et al., 2002; Laurenson et al., 2014). The final concentration of acetone was 1 ppm in control and EE_2_ solutions. The embryos were exposed for 80 days (1–80 days post fertilization) in 2 L tanks in a flow-through system with an exchange rate of 5–6 ml/h. All solutions in the flow-through system were pre-mixed and exchanged daily. Concentrated paramecia culture was fed to each tank once a day for 10 days. Artemia was added to the diet once a day from day 10. *Sera Dry Flakes* (Vipan, Germany) were added to the diet twice daily from week 6. Exposure was terminated at 80 dpf and the fish transferred to clean water were they were kept under normal maintenance conditions until adulthood, resulting in a 120 day remediation period. Sexes were separated based on secondary sexual characteristics at 4 months of age and thereafter re-checked weekly for changes. Animals were kept in a 12/12 h light/dark cycle at 25–27°C, pH 7.0. All experiments and fish handling were performed according to the Swedish Animal Care legislation and approved by the Southern Stockholm Animal Research Ethics Committee (Dnr. S130-09).

### EE_2_ concentrations

Water samples were collected from different tanks at nine separate occasions during the exposure and stored in darkness at −20°C until analysis. EE_2_ concentrations were analyzed in single or duplicate samples as previously described in Volkova et al. (2015b). Briefly, water samples (100 mL) were extracted on 100 mg Strata X-33μ Polymeric Reversed Phase cartridges, reconditioned with MeOH. Dionex ultimate 3000 LC system (Thermo Scientific, San Jose, CA, USA), coupled to a triple quadruple mass spectrometer (TSQ Vantage, Thermo Scientific, San Jose, CA, USA) was used for quantitation of EE_2_ content. The quantification range was 0.5–100 ng/L, using EE_2_-d4 as internal standard.

### Behavior studies

The behaviors were analyzed in the novel tank test (NT; Egan et al., 2009; Figure S1), shoaling test (Moretz et al., 2007; Figure S1), and scototaxis test (Maximino et al., 2010b; Figure S1), as previously described (Volkova et al., 2015b). Briefly, The NT and shoaling tests were performed one after the other in the same test episode. At the right end of the test tank (20 × 20 × 40 cm) filled with 15 L pre-heated tap water, a transparent Plexiglas screen trapped five untreated male or female littermates. A black plastic sheet prevented visual contact with the test compartment. One horizontal and one vertical middle line divided the tank into upper/bottom and right/left halves. The NT test was initiated by introducing a fish to the test tank, and latency time before the first crossing of the horizontal line to the upper half, number of transitions to the upper half and total time in the upper half was recorded. Swimming activity was quantified as number of lines crossed a grid, both horizontally and vertically, during 60 s during the last minute of the NT test. Behavior was videomonitored for 5 min, after which the black screen was removed, revealing the hidden shoal. The shoaling test started when the test fish made contact with the shoal, and latency to leave the shoal (cross the vertical line), number of transitions and time spent in the opposite half of the tank was recorded for 5 min. Fish that did not make contact with the shoal within 5 min were excluded from the analyses.

In the scototaxis test, the test tank (20 × 20 × 40 cm) was divided into one black and one white half and filled with pre-heated tap water up to 10 cm. The tank had two transparent central sliding doors, creating a compartment of 5 × 20 cm. The test fish was introduced into the central compartment, and after a 5 min habituation period the sliding doors were raised and the behavior of the fish was video recorded from above for 5 min. Latency to first entrance into the white half, number of transitions to white half and total time spent in white half was recorded.

All tests were video recorded and manually analyzed. Behavior analyses were not blinded for treatment due to logistical reasons. Three tanks were operating in parallel, and the behavior tests were performed between 9:00 a.m. and 1:00 p.m.

### Dissection and sex verification

Fish previously macroscopically determined as males or females were re-examined based on secondary sexual characteristics, and separated before behavior testing. After behavior testing, two fish of each sex from each family group and treatment group were weighed and then sacrificed by anaesthetization in 0.5‰ 2-phenoxyethanol (Sigma-Aldrich) followed by immediate decapitation. During dissection, gonads and livers were removed and weighed and Gonadosomatic index (GSI) and hepatosomatic index (HSI) were calculated by the formula: organ weight/fish weight × 100. Brains were removed and stored at −80°C in RNA later (Sigma-Aldrich).

### Ion proton RNA sequencing

Based on the results in the novel tank test, RNA sequencing analysis was selected to be performed on control male brains and brains from males exposed to 3 ng/L EE_2_, while for females, brains were from unexposed females and females exposed to 10 ng/L EE_2_. Three biological replicates were used in the RNA sequencing analysis for each treatment group and sex. In the female analysis, fish from the same family groups were represented in both the control and treated samples; meaning that the control females and exposed females were siblings. Male samples were however taken from different family groups and the exposed males were therefore not the siblings of the control males. Brains were homogenized and total RNA extracted with TriReagent (Sigma-Aldrich) according to the manufacturer's protocol. Total RNA from selected samples was submitted to Bea Core Facility (Karolinska Institute, Huddinge, Sweden) for quality checking with R6K Screen Tape. IonProton RNA sequencing was performed by SciLifeLab (Uppsala University, Sweden) collecting ~40 million reads per sample.

The RNA was treated with Ribo-Zero™ rRNA Removal Kit (Epicentre/Illumina) to remove ribosomal RNA, and purified using Agencourt RNAClean XP Kit (Beckman Coulter). The RNA was then treated with RNaseIII according to the Ion Total RNA-Seq protocol (Life Technologies) and purified with Magnetic Bead Cleanup Module (Life Technologies). The size and quantity of RNA fragments were assessed on the Agilent 2100 Bioanalyzer system (RNA 6000 Pico kit, Agilent) before proceeding to library preparation, using the Ion Total RNA-Seq kit (Life Technologies). The libraries were amplified according to the protocol and purified with Magnetic Bead Cleanup Module (Life Technologies). Samples were then quantified using the Agilent 2100 Bioanalyzer system (Agilent) and Fragment Analyzer (Advanced Analytical) and pooled followed by emulsion PCR on the Ion OneTouch™ 2 system using the Ion Proton™ Template OT2 200 v3 Kit (Life Technologies) chemistry. Enriching was conducted using the Ion OneTouch™ ES (Life Technologies). Samples were loaded on an Ion PI v2 Chip (2 samples per chip) and sequenced on the Ion Proton™ System using Ion Proton™ Sequencing 200 v3 Kit (200 bp read length, Life Technologies) chemistry.

### Bioinformatics and biostatistics

The software FastQC (http://www.bioinformatics.babraham.ac.uk/projects/fastqc/) was used to assess read quality, and after that data was mapped to the version 9 of the *Danio rerio* genome using the software Star (Dobin et al., 2013). The mapped reads were converted to count data with the script htseq-count (Anders et al., 2015) using the ensembl annotation of the zebrafish genome sequence. Statistical modeling of gene expression was done with the library edgeR (Robinson et al., 2010) in R (McCarthy et al., 2012) following the workflow suggested by the authors. Before the modeling was done all genes that had expression levels lower than 1 count per million mapped reads in at least three different libraries were omitted from the data set. In the detection of differentially expressed genes family structure were taken into account for the female samples. False discovery rates (FDR) corrected *p*-values were estimated with a Benjamini–Hochberg procedure. After this correction 175 genes had *p*-values lower than 0.05 and were regarded as differentially expressed.

### Functional analysis

Classification of genes and predictions of biological gene function were performed manually, due to the observed low detection capacity of several automated classifications tested. Manual ortholog search was done in Ensembl. GO-terms were found in Zfin (for zebrafish), Entrez gene (for human and rodent orthologs) and NGNC (human orthologs).

### Quantitative real-time PCR (qPCR)

For verification of the RNAseq results, the expression of selected differentially expressed genes in the male brain were analyzed with qPCR. RNA isolation was performed with individual brains from 10 control males and 10 males exposed to 3 ng/L, homogenized in TriReagent (0.8 ml/sample, Sigma-Aldrich, Germany) according to the manufacturer, quantified using NanoDrop ND-1000 spectrophotometer, and RNA quality verified by the 260/280 nm absorption ratio. Reverse transcription was performed from Quantitect® Reverse Transcription Kit (Qiagen) from 1 μg RNA per sample and then diluted 1:10 for qPCR reactions. Bio-Rad C1000 Touch™ Thermal cycler, CFX96™ Real-Time System was used for quantitative Real-Time PCR (qPCR). qPCR reactions were run in triplicates and contained 5 μL cDNA template (25 ng RNA), 2.5 μL each of forward and reverse primer and 10 μL iTaq™ Universal SYBR® Green Supermix (BIO-RAD), using cycling parameters as recommended by the manufacturer. Oligonucleotide primers (Table 1) were designed with Primer-Blast primer designing tool (Ye et al., 2012). Normalized expression was calculated for quantification according to the 2^−ΔCT^ method (Schmittgen and Livak, 2008) with two internal control genes, *18S rRNA* and *Elongation factor 1 alpha (Elf1*α*)*, genes found suitable for zebrafish tissue analysis (Tang et al., 2007).

**Table 1:** **Primer sequences used for qPCR analyses**.

**Gene**	**Primer sequence**
18S r RNA^#^	5′-AATGTCTGCCCTATCAACTTTC-′3
	3′-TGGATGTGGTAGCCGTTTC-′5
Elf1a^*^	5′-TACCCTCCTCTTGGTCGCTTT-′3
	3′-ACCTTTGGAACGGTGTGATTG-′5
Msmo1^*^	5′-TCAGCATCCCTTATGACTGG-′3
	3′-AATGGAGAAGTGAAGTCGTGA-′5
Sqlea^*^	5′-ACAGCTACCAGAGCACTTAAA-′3
	3′-CGCATGTTATACGCATCTCC-′5
Lss^*^	5′-TACAAGCATTTCTTGAGGCAG-′3
	3′-CAGTCAGCAACTATCCAACC-′5
Hmgcs1^*^	5′-CGCTGCTACACTTTACTCCA-′3
	3′-TAGTTTGCTAAATGGTGGGTTTC-′5

# *Filby and Tyler, 2007*.

* *Designed by Primer-BLAST primer designing tool*.

### Statistical analysis

Data from the behavior tests, GSI and HSI, and qPCR was analyzed in the statistical package R (R Core Team, 2013) with generalized linear mixed-effects models using package lme4 (Bates et al., 2015) and followed by *post-hoc* tests where necessary using the multcomp package (Hothorn et al., 2008). To separate maternal family effects from effects of treatment and sex, family was used as random factor as several individuals from the same family where represented at different treatment levels. Sex, treatment and treatment × sex where included as fixed factors. All models were analyzed using LR-tests. In cases were females and males were analyzed separately we used treatment as a fixed factor and family as a random factor. For models with heteroscedastic residuals the response variable was log transformed. Data in the form of counts were analyzed assuming Poisson distribution of residuals.

## Results

### EE_2_ concentrations

The actual concentration of EE_2_ was determined from water samples from 9 occasions during the 80 days exposure period. The determined actual concentrations of EE_2_ (mean ± SEM) in samples taken from the treatment tanks were 2.14 ± 0.33 and 7.34 ± 1.42 ng/L for 3 and 10 ng/L, respectively. This represents 71 and 73% of the nominal concentrations. Control water samples contained no detectable levels (<0.5 ng/L) of EE_2_.

### Body weight, GSI, and HSI

Of the dissected animals all fish phenotypically classified as females contained ovaries, and all fish phenotypically classified as males had testes (*N*_males_ = 47, *N*_females_ = 46). After 120 days of remediation in clean water there were no differences in body weight between control fish and treated fish (Chisq = 5.16, *p* = 0.08). Zebrafish males treated with 3 ng/L EE_2_ had significantly lower GSI compared to control males (*z* = −2.86, *p* = 0.012), while no significant difference was observed in GSI between controls and males treated with 10 ng/L (*z* = −0.214, *p* = 0.98). However, males treated with 10 ng/L had significantly higher HSI compared to control males (*z* = 3.015, *p* = 0.007) whereas males treated with 3 ng/L did not (*z* = 1.66, *p* = 0.22; Table 2). No significant differences in GSI or HSI were found between treated and control females (Table 2).

**Table 2:** **Bodyweight, hepatosomatic index (HSI), and gonadosomatic index (GSI) in male and female zebra fish developmentally exposed to EE_**2**_ after 120 days of remediation. Treatment vs. controls analyzed with Tukey contrasts**.

					**Treatment**		
		***N***	**Mean**	**±SEM**	**Chisq**	***p***	**Tukey**
**MALES**							
Bodyweight (log)					4.852	0.0884	
	Controls	16	0.393	0.018			
	3 ng/L	15	0.367	0.013			−
	10 ng/L	16	0.421	0.018			−
HSI (log)					9.096	0.0106^*^	
	Controls	14	0.709	0.111			
	3 ng/L	15	0.875	0.129			0.221
	10 ng/L	16	1.005	0.101			0.0071^**^
GSI (log)					10.05	0.0066^**^	
	Controls	16	1.558	0.222			
	3 ng/L	15	1.028	0.122			0.0117^*^
	10 ng/L	16	1.51	0.224			0.9750
**FEMALES**							
Bodyweight (log)					1.433	0.4886	
	Controls	16	0.616	0.036			
	3 ng/L	16	0.619	0.038			−
	10 ng/L	14	0.731	0.095			−
HSI (log)					0.489	0.783	−
	Controls	15	2.01	0.298			−
	3 ng/L	16	1.818	0.201			−
	10 ng/L	14	2.115	0.234			−
GSI (log)							−
	Controls	16	12.08	1.168	2.871	0.238	−
	3 ng/L	16	19.86	6.163			−
	10 ng/L	14	14.60	1.866			−

* p < 0.05;

** *p < 0.01*.

### NT test

**Figure 1:**
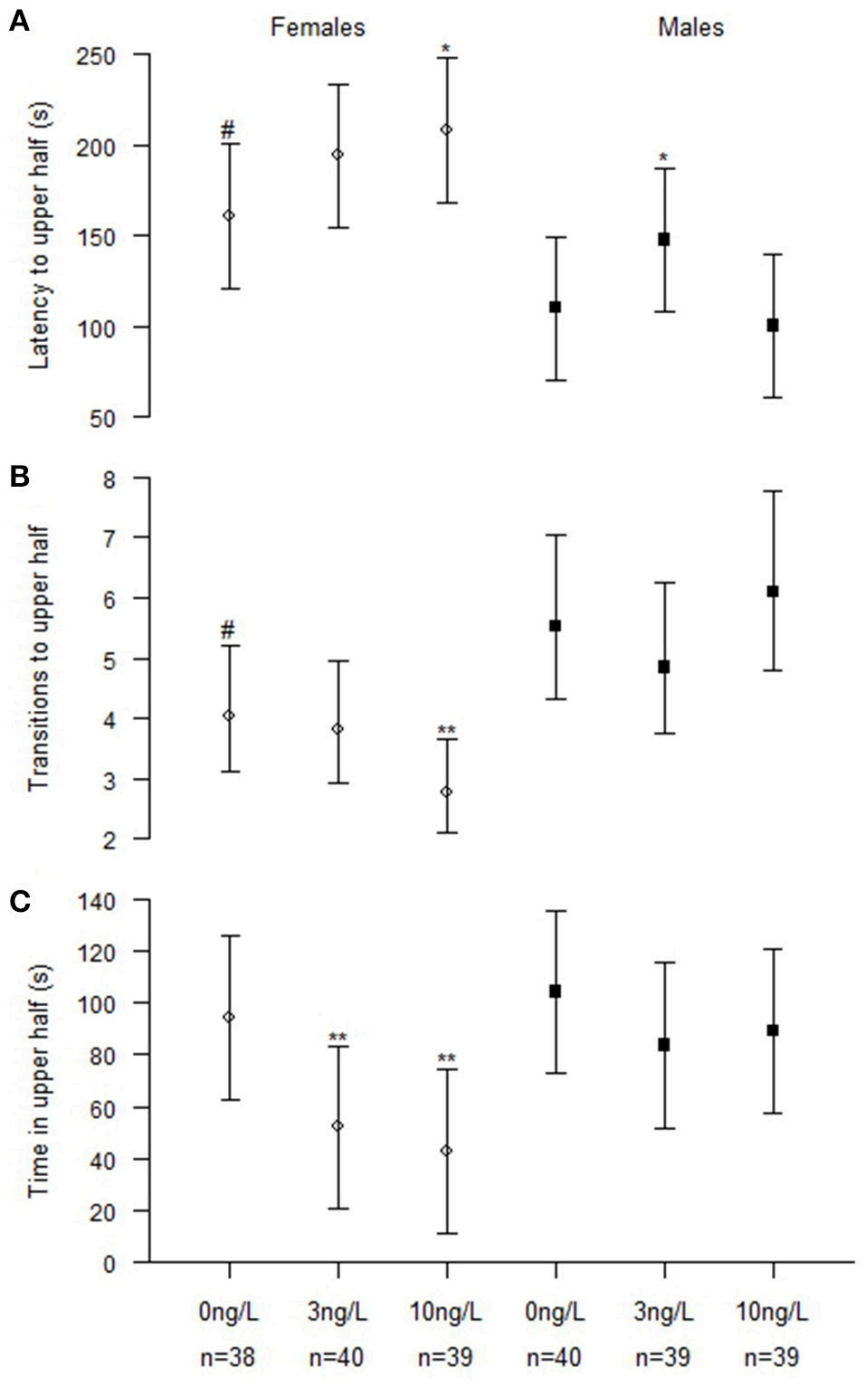
**Novel tank behavior in male and female zebrafish developmentally exposed to 0, 3, or 10 ng/L EE_**2**_ and remediated in clean water for 120 days. (A)** Latency time before crossing the horizontal midline to the upper half. **(B)** Number of transitions to upper half and **(C)** Total time spent in upper half. Data represent mean ± 95% CI, ^#^*p* < 0.05, ^*^p < 0.05, ^**^p < 0.01. ^*^Significantly different from control fishes of the same sex, ^#^control females significantly different from control males.

There were significant interactions between treatment and sex for latency to upper half (Chisq = 7.48, *p* = 0.024), and number of transitions to upper half (Chisq = 14.17, *p* < 0.001). There were no significant interactions found for the time spent in the upper half but it was affected by both treatment (Chisq = 13.77, *p* = 0.001) and sex (Chisq = 12.77, *p* < 0.001; Figure 1, Table 3).

**Table 3:** **Results from behavior tests with mixed effects models following Tukey multiple comparison test when significant treatment effect. Treatment vs. controls analyzed with Tukey contrasts**.

				**Treatment**		**Sex**		**Treatment** × **sex**	
		***N***	**Chisq**	***P***	**Tukey**	**Chisq**	***p***	**Chisq**	***P***
**NOVEL TANK TEST**									
Latency to upper half			7.78	0.021^*^		45.05	<0.001^***^	7.48	0.024^*^
	Controls	78							
	3 ng/L	79			0.139				
	10 ng/L	78			0.022^*^				
Total transitions (log)			1.505	0.471		48.29	<0.001^***^	14.17	<0.001^***^
	Controls	78							
	3 ng/L	79			−				
	10 ng/L	78			−				
Total time in upper half			13.77	0.001^**^		12.77	<0.001^***^	3.39	0.183
	Controls	78							
	3 ng/L	79			0.008^**^				
	10 ng/L	78			<0.001^***^				
**SHOALING TEST**									
Latency to opposite half			0.482	0.79		16.35	<0.001^***^	1.84	0.40
	Controls	57							
	3 ng/L	58			−				
	10 ng/L	51			−				
Total transitions			24.80	<0.001^***^		202.48	<0.001^***^	1.94	0.40
	Controls	57							
	3 ng/L	58			<0.001^***^				
	10 ng/L	51			<0.001^***^				
Time in opposite half			1.45	0.49		45.38	<0.001^***^	2.70	0.26
	Controls	57							
	3 ng/L	58			−				
	10 ng/L	51			−				
**SCOTOTAXIS TEST**									
Latency to cross (log)			5.74	0.057		21.32	<0.001^***^	0.77	0.68
	Controls	69							
	3 ng/L	68			−				
	10 ng/L	79			−				
Total transitions (log)			13.03	0.0015^**^		77.14	<0.001^***^	46.23	<0.001^***^
	Controls	69							
	3 ng/L	68			<0.001^***^				
	10 ng/L	79			0.662				
Time in white zone (log)			6.16	0.046^*^		7.0	0.0008^**^	9.28	0.0097^**^
	Controls	69							
	3 ng/L	68			0.02^*^				
	10 ng/L	79			0.88				

*Analyses include zebra fish males and females treated with EE_2_ during development followed by 120 days remediation. Behaviors were analyzed in the novel tank test, shoaling test and scototaxis test*.

* *p < 0.05*,

** *p < 0.01*,

*** *p < 0.001*.

To further evaluate the effects, we analyzed males (*N*_Control_ = 40, *N*_3 ng_ = 39, *N*_10 ng_ = 39) and females (*N*_control_ = 38, *N*_3 ng_ = 40, *N*_10 ng_ = 39) separately (Figure 1). Females exposed to 3 ng/L spent significantly less time in the upper half (*z* = −2.96, *p* = 0.009). Females exposed to 10 ng/L had significantly increased latency to upper half (*z* = 2.39, *p* = 0.045), fewer transitions to upper half (*z* = −3.0, *p* = 0.008) and spent significantly less time in upper half (*z* = −3.51, *p* = 0.001). Males exposed to 3 ng/L EE_2_ had significantly increased latency to upper half (*z* = 2.50, *p* = 0.034). No significant treatment effects were observed in the novel tank test for males developmentally treated with 10 ng/L compared to the control males (Figure 1).

Swimming activity measured during the last minute of the novel tank test revealed average ± *SD* for females: 70 ± 21, 66 ± 19, 69 ± 15 for 0, 3, and 10 ng/L, respectively, and average ±*SD* for males: 74 ± 25, 70 ± 16 and 69 ± 14 for 0, 3, and 10 ng/L, respectively. The swimming activity was not significantly different between the treatments or between males and females.

### Shoaling test

**Figure 2:**
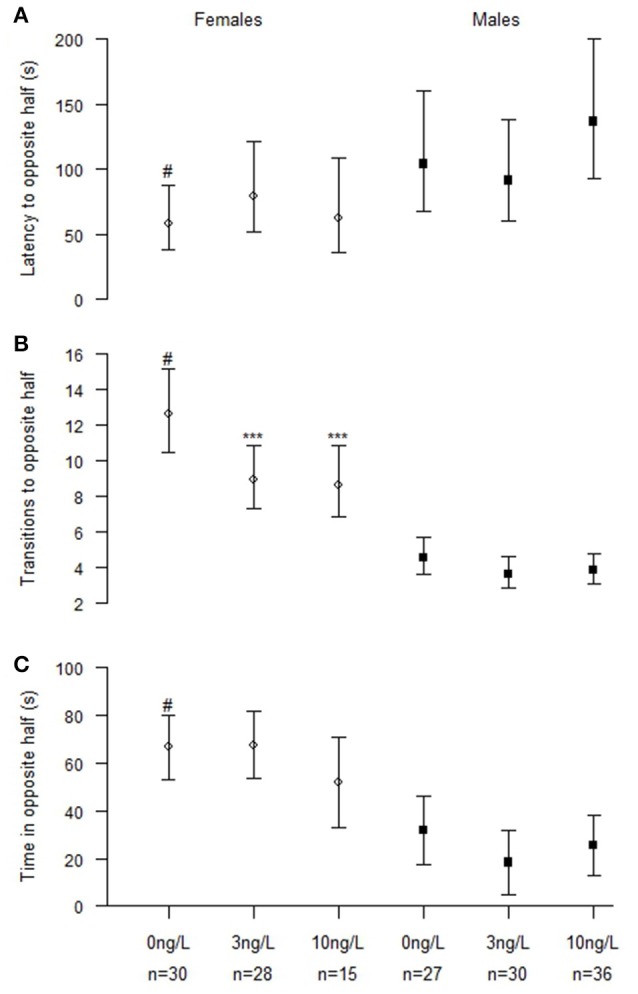
**Shoaling behavior in male and female zebrafish developmentally exposed to 0, 3, or 10 ng/L EE_**2**_ and remediated in clean water for 120 days. (A)** Latency time before leaving shoal (crossing the vertical midline), **(B)** Number of transitions to the opposite half and **(C)** Total time spent in the opposite half. Data represent mean ± 95% CI, ^#^*p* < 0.05, ^***^p < 0.001. ^*^Significantly different from control fishes of the same sex, ^#^control females significantly different from control males.

Fish that failed to make contact with the group within 5 min were excluded from the analyses which slightly lowered the number of observations (N_Control_ = 57, N_3 ng_ = 58, N_10 ng_ = 51). Twenty-one (8 female and 13 male fish), 21 (13 female and 9 male fish), and 27 (24 female and 3 male fish) fishes where excluded for this reason in the 0, 3, and 10 ng/L treatment groups, respectively. EE_2_ exposure significantly decreased the number of transitions made away from shoal (Chisq = 24.8, *p* < 0.001). There were also significant differences by sex in latency to first transition away from shoal (Chisq = 16.3, *p* < 0.001), number of transitions away from shoal (Chisq = 202.5, *p* < 0.001) and total time away from shoal in the opposite half (Chisq = 45.4, *p* < 0.001; Table 3). Even if there were no significant interactions we also analyzed the two sexes separately; males (N_Control_ = 27, N_3 ng_ = 30, N_10 ng_ = 36) and females (N_Control_ = 30, N_3 ng_ = 28, N_10 ng_ = 15; Table 3). Effect of treatment was found only in females. Number of transitions away from the shoal was decreased in females treated with 3 ng/L (*z* = −4.38, *p* < 0.001) and 10 ng/L (*z* = −3.57, *p* < 0.001; Figure 2).

### Scototaxis test

The models including both males and females (N_control_ = 69, N_3 ng_ = 68, N_10 ng_ = 79) reveal significant interactions between sex and treatment in number of entries into white half (Chisq = 46.2, *p* < 0.001) and total time spent in white half (Chisq = 9.28, *p* = 0.0097; Table 3). Latency was not significantly affected by treatment but it was very close (Chisq = 5.75, *p* = 0.057) however there was a significant difference between males and females (Chisq = 21.3, *p* < 0.001; Table 3).

**Figure 3:**
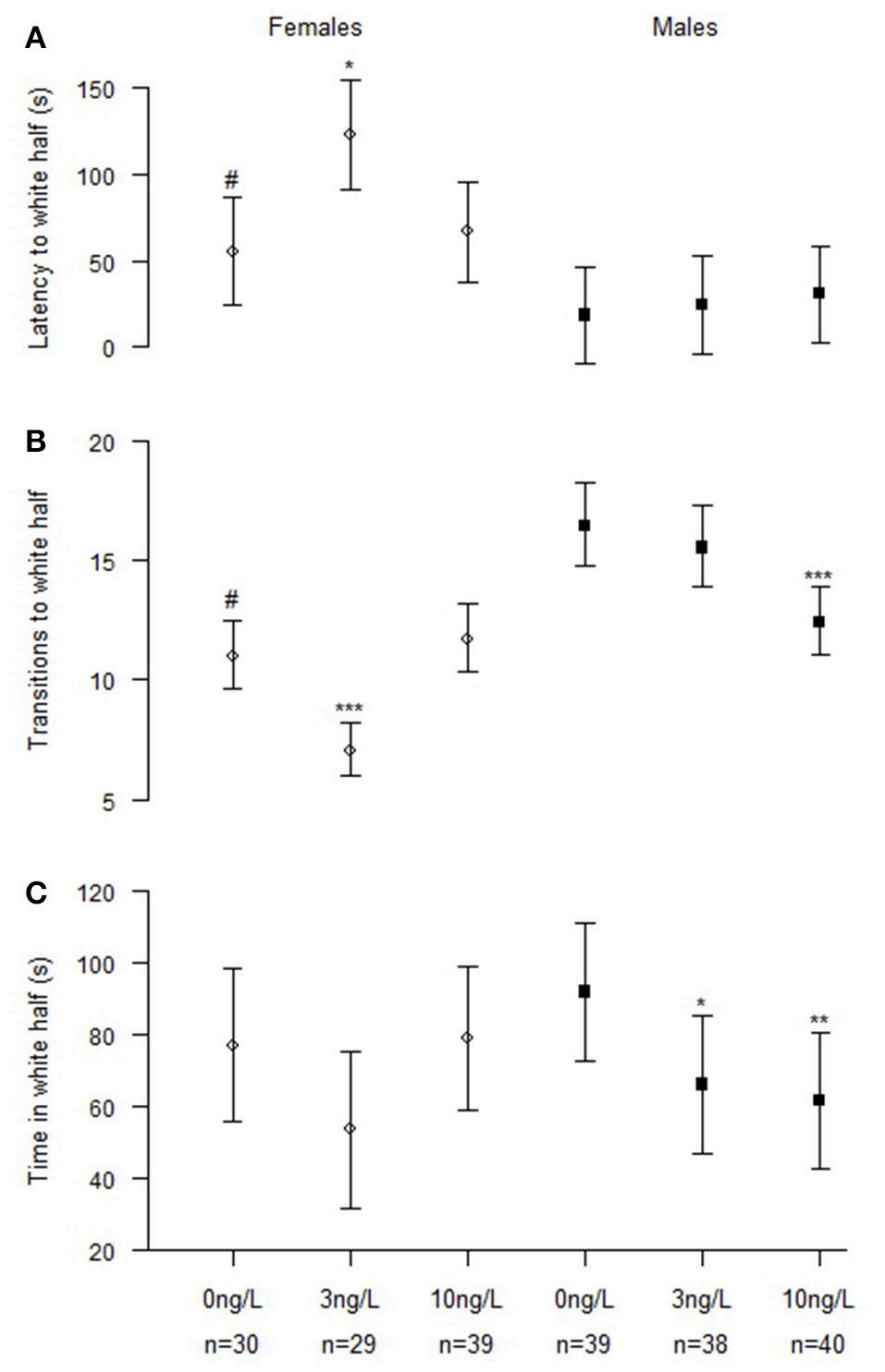
**Scototaxis behavior in male and female zebrafish developmentally exposed to 0, 3, or 10 ng/L EE_**2**_ and remediated in clean water for 120 days. (A)** Latency time before entering the white half, **(B)** Number of transitions to the white half, and **(C)** Total time spent in the white half. Data represent mean ± 95% CI, ^#^*p* < 0.05, ^*^p < 0.05, ^**^p < 0.01, ^***^p < 0.001. ^*^Significantly different from control fishes of the same sex, ^#^control females significantly different from control males.

Analyzing males (N_Control_ = 39, N_3 ng_ = 38, N_10 ng_ = 40) and females (N_Control_ = 30, N_3 ng_ = 29, N_10 ng_ = 39) separately (Figure 3) the results showed that both sexes were affected of treatment. Females exposed to 3 ng/L EE_2_ had increased latency to first transition into white half (*z* = 2.41, *p* = 0.042) and a significantly decreased total number of entries into the white half (*z* = −4.77, *p* < 0.001). No effect was observed in females treated with 10 ng/L EE_2_ compared to control females. Males exposed to 3 and 10 ng/L EE_2_ spent significantly less time in the white half compared to control males (*z* = −2.90, *p* = 0.011) and (*z* = −3.11, *p* = 0.005), respectively. While males exposed to 10 ng/L made significantly fewer entries into the white half (*z* = −4.59, *p* < 0.001) compared to control males.

### RNA sequencing

Of the 33,373 gene features that were annotated in genome sequence, 18,792 in the females, and 18,445 in males had gene expression levels high enough to be retained for analysis. One hundred and forty-six sequences, 95 protein-encoding, and 51 non-coding sequences, were observed to be differentially expressed in brains from developmentally EE_2_-exposed zebrafish after recovery, compared to brains from unexposed controls. In the brain samples from males, 34 coding genes showed significantly different expression levels between control males and males exposed to 3 ng/L EE_2_. For the female brains, 62 coding genes were differentially expressed due to the developmental exposure to 10 ng/L EE_2._ Only one gene was affected by developmental EE_2_ exposure in both male and female brains: *sv2b*, coding for a synaptic vesicle protein, was upregulated by EE_2_ (log fold change: 0.86) in males, while in females the same gene was down-regulated (log fold change: −1.2). In males, another copy of this gene, *svbb*, located upstream of *sv2b* on chromosome 25 was down-regulated by EE_2_ (log fold change: −0.68). No effect on *svbb* was detected in females. The lists of differentially expressed protein coding genes are shown in Table 4 (females) and Table 5 (males). In addition, 48 differently expressed sequences in females and three in males were annotated as non-coding RNA (ncRNA; Table 5).

**Table 4:** **List of protein coding genes significantly affected in brains of female zebrafish developmentally exposed to 10ng/L EE_**2**_ compared to non-exposed fish after 120 days of recovery in clean water**.

**Ensembl ID**	**Gene name**	**Log FC**	**Log CPM**	***p*-value**	**FDR**	**GO term (Zfin)**	**Human ortholog**
**CIRCADIAN RYTM**							
ENSDARG00000057652	dbpb	0.92	6.82	<0.001	<0.001	Regulation of transcription (DNA templated), sequence-specific DNA binding	DBP
ENSDARG00000033160	nr1d1	2.21	5.98	<0.001	<0.001	Regulation of transcription (DNA templated), Sequence-specific DNA binding, steroid and thyroid hormone receptor activity	NR1D1
ENSDARG00000056885	per1a	1.83	6.24	<0.001	<0.001	Photoperiodism, signal transduction, response to hydrogen peroxide	PER1
ENSDARG00000058094	CIART (1 of 2)	1.99	5.54	<0.001	0.003	Not available	CIART
ENSDARG00000088171	CIART (2 of 2)	0.81	6.21	<0.001	0.03	Not available	CIART
ENSDARG00000075397	cipca	1.65	3.91	<0.001	0.004	Not available	CIPC
ENSDARG00000041691	bhlhe41	1.01	7.83	<0.001	0.028	Negative regulation of transcription (DNA templated), protein dimerization, DNA binding	BHLHE41
**CYTOSKELETON AND MOTOR PROTEINS RELATED**							
ENSDARG00000068468	DNAH3	−1.51	5.26	<0.001	<0.001	Not available	DNAH3
ENSDARG00000056888	DNAH8	−1.86	6.06	<0.001	<0.001	Not available	Unknown
ENSDARG00000041723	zgc:55461	−1.48	4.72	<0.001	0.002	Protein polymerization, microtubule-based process, GTP binding, nucleotide binding	TUBB4A/TUBB4B
ENSDARG00000042708	tuba8l	−0.90	4.58	<0.001	0.036	Microtubule-based process, protein polymerization, GTP binding, nucleotide binding	TUBA8
ENSDARG00000087352	AL935046.1	−1.38	5.07	<0.001	0.037	Not available	DNAH2
ENSDARG00000001993	myhb	1.67	3.53	<0.001	0.039	Motor activity, ATP binding, actin binding, Myosin complex	MYH1/13/2/8/4
ENSDARG00000059987	dnah12	−1.10	3.88	<0.001	0.045	Microtubule-based movement, microtubule motor activity	DNAH12
**IMMUNE RESPONSE**							
ENSDARG00000040528	Igals3bpb	1.17	5.37	<0.001	<0.001	Cell adhesion, scavenger receptor activity	LGALS3BP
ENSDARG00000026611	socs3b^*^	−1.21	4.43	<0.001	<0.001	JAK-STAT cascade, protein kinase inhibitor activity	SOCS3
ENSDARG00000053136	b2m	−0.80	6.69	<0.001	0.001	Immune system process, MHCI protein complex	B2M
ENSDARG00000079412	ftr02	−3.20	1.39	<0.001	0.004	Metal ion binding, zinc ion binding	TRIM29
**SYNAPSES**							
ENSDARG00000060711	SV2B	−1.21	4.89	<0.001	0.013	Not available	SV2B
**LIPID TRANSPORT, BINDING, AND METABOLISM**							
ENSDARG00000074749	abca12	−3.08	2.93	<0.001	<0.001	Lipid transport, cholesterol efflux, phospholipid transporter activity	ABCA12
ENSDARG00000039406	prom2	−1.96	4.03	<0.001	0.003	Integral component of membrane	PROM2
ENSDARG00000040295	apoeb	−1.12	7.78	<0.001	0.03	Lipid transport, cholesterol biosynthetic process, negative regulation of neuron apoptotic process	APOE
**CARDIOVASCULAR SYSTEM, BLOOD**							
ENSDARG00000003462	fech	−0.94	5.67	<0.001	<0.001	Erythrocyte maturation, heme biosynthetic process, ferrochelatase activity	FECH
ENSDARG00000031952	mb	3.13	3.31	<0.001	0.002	Vasculogenesis, oxygen transport, response to hypoxia, oxygen binding, heme binding	MB
ENSDARG00000055101	hmox2a	−0.72	5.41	<0.001	0.008	Hemeoxygenase (decyclizing) activity, oxidation-reduction process, heme oxidation	HMOX2
ENSDARG00000078529	Bai1b	0.82	8.61	<0.001	0.04	Regulation of angiogenesis	BAI1
**CELL ADHESION**							
ENSDARG00000089195	pcdh2g20	−1.90	2.10	<0.001	0.02	Cell adhesion, calcium ion binding	Unknown
ENSDARG00000014047	cldn7b	−2.32	2.87	<0.001	0.04	Structural molecular activity	CLDN7
**CHROMATIN REMODELING**							
ENSDARG00000092933	cbx8a	−4.59	1.32	<0.001	<0.001	Nucleus	CBX8
ENSDARG00000060645	sirt7	−1.34	4.49	<0.001	0.004	NAD+ binding	SIRT7
**POST-TRANSCRIPTIONAL MODIFICATIONS**							
ENSDARG00000051970	smu1b	1.21	6.44	<0.001	0.010	Not available	SMU1
**APOPTOSIS**							
ENSDARG00000063572	perp	−0.91	5.07	<0.001	0.015	Regulation of apoptotic process, response to UV	PERP
**THYROID**							
ENSDARG00000020084	tg	−2.62	1.93	<0.001	0.004	Carboxylic ester hydrolase activity	TG
**SIGNAL TRANSDUCTION TRANSMEMBRANE RECEPTOR**							
ENSDARG00000002494	itgb6	−2.29	1.70	<0.001	0.018	Integrin-mediated signaling pathway, cell adhesion, receptor activity	ITGB6
ENSDARG00000016470	anxa5b	−2.15	3.06	<0.001	0.02	Negative regulation of coagulation, regulation of cell motility, calcium-dependent phospholipid binding	ANXA5
ENSDARG00000026611	socs3b^*^	−1.21	4.43	<0.001	<0.001	JAK-STAT cascade, protein kinase inhibitor activity	SOCS3
**OTHER**							
ENSDARG00000092115	eif4a1a	−1.18	8.96	<0.001	<0.01	Translational initiation, nucleotide binding, ATP binding, helicase activity, hydrolase activity	SENP3, EIF4A1
ENSDARG00000091245	dnajc7	−1.22	4.84	<0.001	<0.001	Not available	DNAJC7
ENSDARG00000009978	icn	−1.91	4.54	<0.001	0.03	Calcium ion binding	S100A2/3/4/5/6
ENSDARG00000069476	spint2	−3.43	3.45	<0.001	0.01	Serine-type endopeptidase inhibitor activity	SPINT2
ENSDARG00000024195	znf395b	0.95	5.74	<0.001	0.01	Metal ion binding	ZNF395
ENSDARG00000068923	unmodl1	−2.20	2.49	<0.001	0.019	Peptidase inhibitor activity, calcium ion binding	UMODL1
ENSDARG00000075984	hsbp1l1	−2.53	1.34	<0.001	0.025	Not available	HSBP1
ENSDARG00000038729	s100z	−1.53	2.97	<0.001	0.038	Metal ion binding, calcium binding	S100Z
ENSDARG00000074807	tbrg4	−0.75	4.65	<0.001	0.048	Protein kinase activity	TBRG4
ENSDARG00000052012	rtn4rl2a	1.09	5.51	<0.001	0.042	Not available	RTN4RL2
**UNKNOWN**							
ENSDARG00000089382	zgc:158463	−3.63	7.93	<0.001	<0.001	Not available	Unknown
ENSDARG00000088436	CT956064.3	−3.58	5.84	<0.001	<0.001	Not available	Unknown
ENSDARG00000091744	BX296557.7	−2.97	7.17	<0.001	<0.001	Not available	Unknown
ENSDARG00000031588	si:dkey-239b22.1	−4.22	3.44	<0.001	<0.001	Cell-matrix adhesion	Unknown
ENSDARG00000068374	si:ch211-132b12.7	1.50	6.56	<0.001	<0.001	Not available	Unknown
ENSDARG00000091234	CU019646.2	−2.25	2.30	<0.001	0.001	Not available	Unknown
ENSDARG00000067703	NLRP	1.32	4.62	<0.001	0.0037	Not available	Unknown
ENSDARG00000079175	si:ch211-79k12.1	−2.44	2.44	<0.001	0.004	Not available	Unknown
ENSDARG00000088142	Unknown	1.72	4.74	<0.001	0.004	Not available	Unknown
ENSDARG00000087067	CABZ01073069.1	3.73	0.84	<0.001	0.007	Not available	Unknown
ENSDARG00000088938	BX548011.4	−3.12	1.34	<0.001	0.008	Not available	Unknown
ENSDARG00000036700	Unknown	−1.71	5.00	<0.001	<0.01	Not available	Unknown
ENSDARG00000093780	si:ch211-212c13.6	−2.42	3.63	<0.001	<0.01	Not available	Unknown
ENSDARG00000042829	si:dkey-30j22.1	−2.46	3.73	<0.001	0.019	Calcium ion binding, extracellular matrix structural constituent	Unknown
ENSDARG00000070212	zgc:158463	−1.69	9.23	<0.001	0.03	Not available	Unknown
ENSDARG00000080675	si:dkey-71b5.7	−2.71	3.70	<0.001	0.019	Not available	Unknown
ENSDARG00000090930	si:ch211-120g10.1	−1.93	4.54	<0.001	<0.001	Not available	Unknown

* *This gene is assosciated with multiple categories LogFC, Log fold change; Log CPM, Log count per million; FDR, False discovery rate. Human orthologs were found in Ensembl and categories assigned manually based on information available on Entrez gene, NGNC, and Omim databases. GO-terms were found in Zfin*.

**Table 5:** **List of protein coding genes significantly affected in brains of male zebrafish developmentally exposed to 3ng/L EE_**2**_ compared to non-exposed fish after 120 days of recovery in clean water**.

**Ensembl ID**	**Gene name**	**Log FC**	**Log CPM**	***p*-value**	**FDR**	**GO term (Zfin)**	**Human ortholog**
**CHOLESTEROL BIOSYNTHESIS**							
ENSDARG00000052738	hmgcs1	1.01	5.75	<0.001	0.01	Oligodendrocyte development, isoprenoid biosynthetic process	HMGCS1
ENSDARG00000079946	sqlea	1.84	4.21	<0.001	0.01	Fatty acid biosynthesis process, metabolic process, oxidation reduction process, 3-hydroxyacyl-CoA dehydrogenase activity, flavin adenine dinucleotide binding, oxidoreductase activity, squalene monooxygenase activity	SQLE
ENSDARG00000061274	lss	1.34	3.95	<0.001	0.02	Baruol synthase activity	LSS
ENSDARG00000055876	msmo1	0.98	5.08	<0.001	0.01	Fatty acid biosynthesis process, endoplasmic reticulum, C-4 methylsterol oxidase activity	MSMO1
**SYNAPSES**							
ENSDARG00000060711	SV2B	0.86	5.25	<0.001	0.01	Not available	SV2B
ENSDARG00000090540	Svbb	−0.68	5.47	<0.001	0.02	Transmembrane transport, integral component of membrane, transmembrane transporter activity	SV2B
ENSDARG00000078624	arhgef9b^*^	−1.05	5.27	<0.001	0.005	Regulation of Rho protein signal transduction, Rho guanyl-nucleotide exchange factor activity	ARHGEF9
**DNA REPAIR**							
ENSDARG00000079015	brca2	1.20	4.02	<0.001	0.01	DNA repair, regulation of transcription, female gonad development, spermatogenesis, centrosome, γ-tubulin binding	BRCA2
ENSDARG00000055754	smc1a	1.01	6.22	<0.001	0.01	DNA repair, chromosome organization, ATP binding, chromatin binding	SMC1A
**IMMUNE RESPONSE**							
ENSDARG00000024877	ptgr1	−2.32	4.24	<0.001	0.015	Oxidation-reduction process, oxidoreductase activity, zinc ion binding	PTGR1
ENSDARG00000070396	serpinb1l2	5.71	1.11	<0.001	<0.001	Negative regulation of endopeptidase activity, extracellular space, serine-type endopeptidase activity	SERPINB1
**CARDIO VASCULAR SYSTEM AND BLOOD**							
ENSDARG00000070449	tspan5b	−2.02	2.88	<0.001	0.002	Integral component of membrane	TSPAN5
ENSDARG00000097011	hbaa1	1.48	6.56	<0.001	0.005	Response to hypoxia, hemoglobin complex, heme binding	HBA1
ENSDARG00000069735	si:ch211-5k11.6	2.79	2.01	<0.001	0.008	Oxygen transport, hemoglobin complex, heme binding	Unknown
**SKIN AND CONNECTIVE TISSUE**							
ENSDARG00000078322	col12a1a	0.94	5.00	<0.001	0.019	Collagen trimer	COL12A1
ENSDARG00000033760	pmelb	1.90	2.62	<0.001	0.005	Not available	PMEL
**POST-TRANSCRIPTIONAL MODIFICATIONS**							
ENSDARG00000087678	rbm22	2.46	1.52	<0.001	0.04	RNA splicing, multicellular organismal development	RBM22
**SIGNAL TRANSDUCTION**							
ENSDARG00000022185	RGL3	−1.58	2.51	<0.001	0.03	Not available	RGL3
ENSDARG00000086838	ITGA2	−3.48	1.33	<0.001	0.007	Not available	ITGA2
ENSDARG00000078624	arhgef9b^*^	−1.05	5.27	<0.001	0.005	Regulation of Rho protein signal transduction, Rho guanyl-nucleotide exchange factor activity	ARHGEF9
**OTHER**							
ENSDARG00000092281	flnb	−0.93	4.35	<0.001	0.04	Not available	FLNB
ENSDARG00000009524	rnf150b	0.63	7.20	<0.001	0.01	Metal ion binding, zinc ion binding	RNF150
ENSDARG00000077799	EGR4	1.22	5.50	<0.001	0.015	Not available	EGR4
ENSDARG00000041294	noxo1a	−2.65	1.36	<0.001	0.002	Phosphatidylinositol binding	NOXO1
ENSDARG00000042988	SLC24A2 (1 of 2)	−3.50	2.95	<0.001	0.009	Not available	SLC24A2
ENSDARG00000058508	CFAP70	−2.48	4.29	<0.001	0.01	Not available	CFAP70
**UNKNOWN**							
ENSDARG00000094587	si:dkey-35m8.1	−0.87	4.67	<0.001	0.017	Not available	Unknown
ENSDARG00000070516	si:dkeyp-52c3.2	4.74	3.86	<0.001	<0.001	GTP binding	Unknown
ENSDARG00000070506	CR450842.1	6.58	1.17	<0.001	<0.001	Not available	Unknown
ENSDARG00000093998	si:ch73-7i4.2	−7.47	1.90	<0.001	<0.001	Not available	Unknown
ENSDARG00000068621	si:ch211-181d7.3	0.99	6.44	<0.001	<0.001	Not available	Unknown
ENSDARG00000089831	NLRP6	−1.21	4.66	<0.001	<0.001	Not available	Unknown
ENSDARG00000068621	si:ch211-181d7.3	0.99	6.44	<0.001	<0.001	Not available	Unknown
ENSDARG00000089582	si:dkey-265e15.2	2.75	1.19	<0.001	<0.001	Not available	Unknown
ENSDARG00000091847	si:ch211-181d7.1	0.84	5.85	<0.001	0.01	ATP binding, nucleotide binding	Unknown

* *This gene is associated with multiple categories. logFC, Log fold change; logCPM, Log count per million; FDR, False discovery rate. Human orthologs were found in Ensembl and categories assigned manually based on information available on Entrez gene, NGNC, and Omim databases. GO-terms were found in Zfin*.

### Functional analysis of protein coding genes

Functional analyses were performed for males and females separately. As several automated classifications tested yielded low percentage of genes classified to function, a manual search was performed based on information from human and rodent ortholog data offered through Entrez gene and NGNC databases in addition to the zebrafish database Zfin. This search identified 73% of all differentially expressed genes (45 out of 62 genes for females and 25 out of 34 genes for males). Differentially expressed genes in Tables 4, 5 are presented under the categories that were manually assigned. For female brains, the search identified “Circadian Rhythm” and “Cytoskeleton and motor proteins” as top putative biological pathways, with seven affected genes each (Table 4). “Cholesterol biosynthesis” was identified as the top putative pathway in male brains, with four differentially expressed genes (Table 5).

#### Circadian rhythm

A manual homolog search in Ensembl identified a total of seven differentially expressed genes encoding proteins presumably involved in the circadian rhythm in brains from developmentally EE_2_-exposed compared with unexposed female brains (Table 4). The genes were *dbpb* (log fold change: 0.92), *nr1d1* (log fold change: 2.21), *per1a* (log fold change: 1.83), *ciarta* (log fold change: 1.99), *ciartb* (log fold change: 0.81), *cipca* (log fold change: 1.65), and *bhlhe41* (log fold change: 1.01). None of the differentially expressed genes in male brains were associated with the circadian rhythm.

#### Cholesterol biosynthesis and transportation

Cholesterol biosynthesis was identified as the top putative pathway affected by developmental EE_2_-exposure in zebrafish male brains after 120 days of remediation (Table 5). Four genes, *sqlea* (log fold change: 1.84), *lss* (log fold change: 1.34), *msmo1* (log fold change: 0.98), and *hmgcs1* (log fold change: 1.01) were significantly upregulated by EE_2_. None of these genes were significantly altered by EE_2_ exposure in female brains. However, the manual search identified three genes involved in binding and transporting cholesterol (Table 3) which were significantly down-regulated in brains of females developmentally exposed to EE_2_ compared with brains from control females: *apoeb* (log fold change: −1.12), *abca12* (log fold change; −3.08), and *prom2* (log fold change: −1.96).

#### Synapses

Several genes involved in synaptogenesis and synapse function were affected by developmental EE_2_ exposure in both male and female zebrafish brains. In male brains, in addition to the altered expression of the two synaptic vesicle genes mentioned above, *sv2b* (log fold change: 0.86) and *svbb* (log fold change: −0.68), also a decreased expression of *arhgef9b* (log fold change: −1.05) encoding collybistin was observed in response to developmental EE_2_ exposure (Table 5). Females developmentally exposed to EE_2_ showed a down-regulation of brain *sv2b* expression (log fold change: −1.20) as mentioned above, and also of the expression *si:ch211-120g10.1* (log fold change: −1.93) (Table 4). In addition, females showed an upregulation of brain *rtn4rl2a* (log fold change: 1.08) and *bai1b* expression (log fold change: 0.82), coding for a negative regulator of angiogenesis in the brain, in response to EE_2_.

#### Cytoskeleton and motor proteins

Developmental EE_2_ exposure affected seven genes related to the cytoskeleton and motor proteins in zebrafish female brains (Table 4). The expression of four axonemal dynein genes were significantly down-regulated; *dnah3* (log fold change: −1.51), *dnah8* (log fold change −1.86), *dnah12* (log fold change: −1.10) and *AL935046.1*, a suggested ortholog of the human DNAH2 (log fold change: −1.38). Down-regulated expression of two tubulin genes, *tuba8l* (log fold change: −0.90) and *zgc:55461*, a suggested ortholog to the human *TUBB4A* and *TUBB4B* genes (log fold change: −1.48), was also detected. Expression of the myosin complex-encoding gene *myhb* (log fold change: 1.67) was upregulated in the brains of females developmentally exposed to EE_2_. No genes related to cytoskeleton or motor proteins were found to be affected in male brains.

#### Heme metabolism and cardiovascular system

Several genes coding for heme-binding proteins were affected by developmental EE_2_ treatment in both male and female zebrafish. In male brains, EE_2_ exposure during early life resulted in an upregulation of *hbaa1* (log fold change: 1.48) and *si:ch211-5k11.6* (log fold change: 2.79) expression (Table 5); both gene products are predicted to be part of the hemoglobin complex. In female brains there was an upregulation of the *mb* gene expression (log fold change: 3.13) encoding myoglobin, by EE_2_ (Table 4). Further, a down regulation of *fech* expression, encoding the terminal enzyme of the heme biosynthesis pathway (log fold change: −0.94) as well as of *hmox2a* (log fold change: −0.72) was observed. Also *bai1b* expression (log fold change: 0.82), a negative regulator of angiogenesis, was upregulated after developmental EE_2_ exposure followed by 120 days of remediation (Table 4), while in males, the expression of *tspan5b*, coding for a protein suggested to be involved in angiogenesis, was down-regulated (log fold change: −2.02, Table 5).

#### Immune system

The brain expression of several genes in different ways involved in the immune response and inflammation were found to be affected by developmental exposure to EE_2_. The macrophage associated lectin *lgals3bpb* (log fold change: 1.17) gene expression was upregulated in female brains by the exposure, while the expression of *socs3b* (log fold change: −1.21), *b2m* (log fold change: −0.80), and *ftr02f* (log fold change: −3.20) were down-regulated (Table 4). In males, downregulation of the expression of *ptgr1* (log fold change: −2.32), encoding a protein involved in the inactivation of the pro-inflammatory factor leukotriene B4, and an upregulation of *serpinb1l2* (log fold change: 5.71) expression was observed in zebrafish male brains by EE_2_ (Table 5).

#### DNA repair and chromatin remodeling

The brain expression of two genes involved in DNA repair and recombination, *brca2* (log fold change: 1.20) and *smc1a* (log fold change: 1.01), were upregulated in the brains of zebrafish males developmentally exposed to EE_2_ (Table 5). These genes were not affected in developmentally exposed female zebrafish. Females showed a marked down-regulation of brain *cbx8a* (log fold change: −4.59) and also of *sirt7* (log fold charge: −1.34) expression by EE_2_ (Table 4), encoding two proteins believed to be involved in chromatin remodeling and DNA repair.

#### Non-coding sequences

Of the differentially expressed sequences 48 of the 110 in the females and 3 of 34 in males were annotated as non-coding RNA (ncRNA). A significant fraction, 19 of the 48 ncRNA in the female brain were novel microRNAs (miRNAs) whilst none of the 3 in males were miRNA (Table 6).

**Table 6:** **List of non-coding genes significantly affected in brains of zebrafish developmentally exposed to 3 ng/L (males) and 10 ng/L (females) EE_**2**_ compared to non-exposed fish after 120 days of recovery in clean water**.

**Ensembl ID**	**Log FC**	**Log CPM**	***P*-value**	**FDR**	**Chromosome**	**Human ortholog**
**FEMALES**						
**Novel miRNA**						
ENSDARG00000088865	−3.62	5.46	<0.001	<0.001	5	Unknown
ENSDARG00000088533	−3.87	5.16	<0.001	<0.001	5	Unknown
ENSDARG00000088976	−3.54	3.17	<0.001	<0.001	5	Unknown
ENSDARG00000087432	−3.41	6.30	<0.001	<0.001	5	Unknown
ENSDARG00000088430	−3.34	4.26	<0.001	<0.001	5	Unknown
ENSDARG00000087068	−4.47	3.42	<0.001	<0.001	5	Unknown
ENSDARG00000089384	−3.42	2.91	<0.001	<0.001	20	Unknown
ENSDARG00000086686	−3.77	2.17	<0.001	<0.001	20	Unknown
ENSDARG00000088510	−2.61	4.08	<0.001	<0.001	5	Unknown
ENSDARG00000084533	−3.39	2.86	<0.001	<0.001	20	Unknown
ENSDARG00000084962	−2.78	2.46	<0.001	<0.001	20	Unknown
ENSDARG00000090280	−2.58	2.30	<0.001	<0.001	20	Unknown
ENSDARG00000090175	−3.33	4.74	<0.001	<0.001	5	Unknown
ENSDARG00000091738	−3.15	2.00	<0.001	<0.001	20	Unknown
ENSDARG00000090733	−3.66	2.16	<0.001	<0.001	5	Unknown
ENSDARG00000088313	−2.99	2.40	<0.001	0.0035	20	Unknown
ENSDARG00000088673	−3.04	1.82	<0.001	0.0190	20	Unknown
ENSDARG00000087315	−2.87	2.43	<0.001	0.0425	20	Unknown
ENSDARG00000088865	−3.62	5.46	<0.001	<0.001	5	Unknown
**Novel snoRNA**						
ENSDARG00000082087	−1.18	5.75	<0.001	<0.001	7	SNORD31
ENSDARG00000082611	−1.15	6.17	<0.001	<0.001	7	SNORA23
ENSDARG00000083108	−1.25	5.64	<0.001	<0.001	20	Yes, several
ENSDARG00000083400	−0.81	6.17	<0.001	<0.001	20	Yes, several
ENSDARG00000083784	−1.34	4.65	<0.001	0.0013	7	SNORD22
ENSDARG00000082008	−2.22	5.61	<0.001	0.0013	10	SNORD14C
ENSDARG00000081115	1.11	8.63	<0.001	0.0059	23	Yes. several
ENSDARG00000080942	−1.80	4.31	<0.001	0.0071	2	SNORD66
ENSDARG00000081931	−0.71	6.32	<0.001	0.0086	19	Yes, several
ENSDARG00000084828	−0.91	7.09	<0.001	0.0107	4	Yes, several
ENSDARG00000081849	−1.04	4.22	<0.001	0.0112	1	Yes, several
ENSDARG00000082928	−1.16	4.38	<0.001	0.0137	5	Yes, several
ENSDARG00000084628	−1.54	5.55	<0.001	0.0251	20	SNORA27
ENSDARG00000083378	−1.11	4.51	<0.001	0.0327	5	Yes, several
ENSDARG00000081235	−0.77	6.73	<0.001	0.0398	10	SNORD14D
**Novel snRNA**						
ENSDARG00000083063	−1.29	6.19	<0.001	<0.001	8	Unknown
ENSDARG00000083455	−3.34	0.52	<0.001	0.0024	4	Yes, several
ENSDARG00000082472	4.33	8.56	<0.001	0.0046	13	Unkown
**Novel rRNA**						
ENSDARG00000091828	−6.47	5.23	<0.001	<0.001	5	Yes, several
ENSDARG00000091776	−5.69	4.92	<0.001	<0.001	5	Yes, several
ENSDARG00000090705	−5.03	5.21	<0.001	0.0086	4	Yes, 2
**Novel Mt rRNA**						
ENSDARG00000083480	−5.15	7.24	<0.001	<0.001	MT	Unknown
ENSDARG00000082084	−2.39	4.73	<0.001	0.0135	MT	Unknown
ENSDARG00000082753	−1.68	11.74	<0.001	0.0142	MT	Unknown
**Known antisense**						
ENSDARG00000091988	−3.25	0.27	<0.001	0.0196	19	Unknown
**Known lincRNA**						
ENSDARG00000096403	−2.20	3.92	<0.001	<0.001	20	Unknown
**Known processed transcript**						
ENSDARG00000095598	−1.39	2.68	<0.001	0.0260	9	Unknown
**MALES**						
**Novel Ribozyme**						
ENSDARG00000084661	−1.54	4.36	<0.001	<0.001	2	Unknown
**Novel snRNA**						
ENSDARG00000080300	6.56	9.75	<0.001	0.018	7	Yes, several
**Novel snoRNA**						
ENSDARG00000084622	0.92	8.14	<0.001	0.046	15	Yes, several

*Human orthologs were found in Ensembl*.

### Verification of differential expression by qPCR

**Figure 4:**
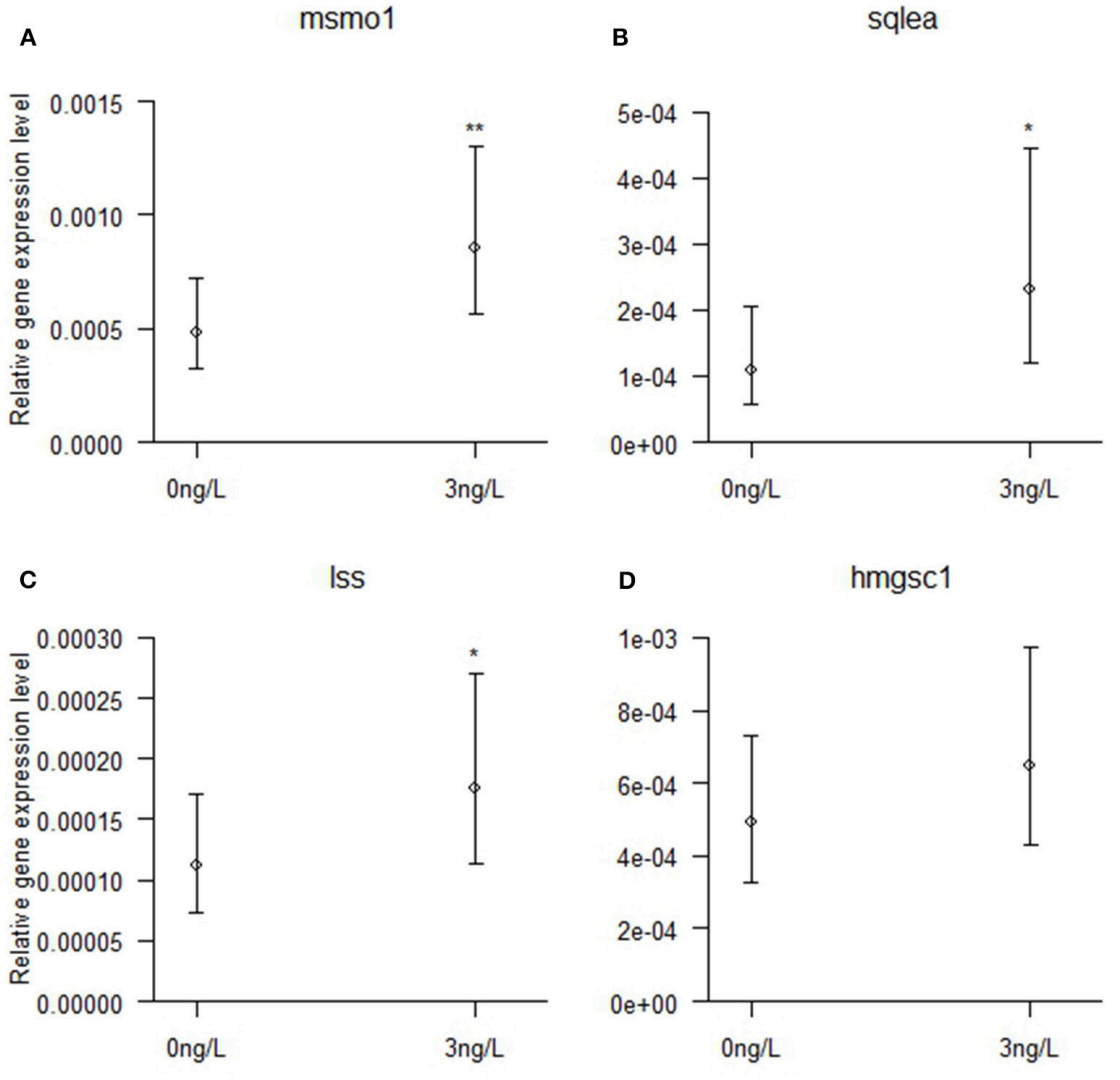
**Brain mRNA expression of (A)** *msmo1*, **(B)** *sqlea*, **(C)** *lss*, and **(D)** *hmgcs1* in zebrafish males developmentally exposed to 0 or 3 ng/L EE_2_ and remediated in clean water for 120 days. The mRNA expression was quantified with qPCR and normalized against the expression of *18S RNA* and *Elf1a*, displaying the relative gene expression. Data represent mean ± 95% CI of 10 samples/group, ^*^p < 0.05, ^**^p < 0.01. ^*^Significantly different from control group.

Verification of the differential gene expression observed by RNA sequencing analysis was performed by qPCR for four selected target genes in the cholesterol biosynthesis pathway that was differentially upregulated by 3 ng/L EE_2_ in male brains. Ten brain samples each in exposure and control groups were analyzed. As shown in Figure 4, significant increased expression was observed for *msmo1* (Chisq = 9.00, df = 1, *p* = 0.003), *sqlea* (Chisq = 6.41, df = 1, *p* = 0.011), and *lss* (Chisq = 4.94, df = 1, *p* = 0.026). The expression of *hmgsc1* was however not significantly higher in brains from EE_2_-exposed males than in control brains (Chisq = 1.86, df = 1, *p* = 0.173).

## Discussion

The present study verifies and extends our previous findings (Volkova et al., 2015b) that developmental exposure of zebrafish to EE_2_ increase anxiety and shoal cohesion as adults. We also present an analysis of the whole brain transcriptome in exposed and control fish by RNAseq in an attempt to understand the mechanisms of the persistent behavior phenotype. The transcriptome analysis revealed marked differences in gene expression in males and females, with 94 protein coding genes significantly affected by the developmental exposure to EE_2_ (Tables 4, 5).

We used the same nominal concentrations as in Volkova et al. (2015b), but the actual measured concentrations were higher in the present study due to differences in exposure protocol. Although the experimental procedures were not identical, and the source of the AB strain was different among the studies, the similarities in the behavior results are obvious. Clear anxiogenic effects of EE_2_ on behavior were observed in both the NT test and scototaxis test (Figures 1, 3). Both tests have been characterized by means of pharmacologic drugs in zebrafish (Maximino et al., 2015) however, only when exposed as adults. Scototaxis and NT have been suggested to be complementary rather than identical tests for anxiety based on differences in stimuli (Maximino et al., 2010a; Blaser and Rosemberg, 2012). Also, novelty in the NT has been suggested to be a conditional stimulus, and as such might be sensitive to minor external factor fluctuations, as discussed in Maximino et al. (2010a) and Blaser and Rosemberg (2012).

The current study verified that developmental EE_2_ exposure of zebrafish increases anxiety as adults, after a long remediation period in clean water. Increased anxiety is well-established as an EDC effect in mammals (Dugard et al., 2001; Ryan and Vandenbergh, 2006; Gioiosa et al., 2007; Skinner et al., 2008; Gonçalves et al., 2010), although the outcome differs by sex, age at treatment, exposure dose, and length (Gioiosa et al., 2013). Anxiety due to EE_2_ has been observed by us and others in several fish species (Hallgren et al., 2011; Reyhanian et al., 2011; Heintz et al., 2015). The behaviors affected are of great importance for fitness of fish populations in the wild, affecting survival, and predator avoidance, but also mating success and foraging.

In the shoaling test, developmentally exposed fish ventured away from the shoal less in agreement with previous results (Volkova et al., 2015b). The effects of EDCs on shoaling behavior are unclear at present, both increased, decreased, and unaffected shoal cohesion have been shown in EDC-exposed fish (Espmark Wibe et al., 2002; Ward et al., 2006; Xia et al., 2010; Reyhanian et al., 2011). A stress component in the behavior is expected, as tight shoaling is an anti-predator strategy, but no characterization of the test system by means of anxiogenic and anxiolytic drugs have been performed, however such studies are needed.

To learn more about what changes in the brain could be discerned to accompany the behavior phenotype, we performed RNA sequencing of the brain transcriptome. We acknowledge that this transcriptome analysis is hampered by the few biological replicates, and conclusions from the data have to be drawn with caution. Also, the use of whole brains might hide region-specific alterations. Transcriptome data are challenging to interpret, and we can only speculate on connections between identified pathways and imprinting of increased stress sensitivity due to developmental EE_2_ exposure. However, RNA sequencing analyses of zebrafish brain are still few, and this study represents, to the best of our knowledge, the first study of estrogenic effects induced during development and persisting as a behavior phenotype in the adult fish. Further studies are needed to support, or contradict, the current findings.

No effects were observed in genes involved in the stress axis, neither on CRH, related genes in the hypothalamo-pituitary-interrenal (HPI) axis nor the monoamine system shown to be involved in their regulation. We did, however, identify some possible candidate pathways that might indirectly affect anxiety behavior. It is thus possible that the alterations in anxiety behavior induced during development are not mediated via the most obvious target genes in the brain. The connection between estrogens, stress and the regulation of HPI axis and monoaminergic system in fish is well-established (Winberg and Nilsson, 1993; Pottinger et al., 1996; Winberg et al., 1997; Carpenter et al., 2007; Egan et al., 2009). Previous studies observed that acute EE_2_ exposure result in altered expression of genes involved in the glucocorticoid receptor signaling pathway in the pituitary of sub-adult female Coho salmon (Harding et al., 2013) as well as in the hepatic tryptophan metabolism pathway, a precursor to serotonin, in adult zebrafish (Wit et al., 2010). The fish in this study were, however, not under EE_2_ exposure, nor were they subjected to stressors prior to tissue collection, thus decreasing the likelihood of acute effects on the stress axis. The current study does, however, not support that direct effects on stress signaling underlie the adult anxiety phenotype observed in this study.

Only one gene, *sv2b*, was affected in both sexes of the treated animals. No other genes were significantly altered in both sexes after recovery from developmental EE_2_ exposure, though some similarities between the affected pathways exist. Both males and females had alterations in pathways related to the synapses, immune response, lipid metabolism, and heme biosynthesis/degradation, even if the affected genes within these pathways were not identical. As the behavior phenotype is similar, this might suggest that different mechanisms mediate the anxiety phenotype in males and females.

The most significantly affected pathway in females was the circadian rhythm (Table 4) with differential expression of seven genes in response to EE_2_. Among them were *per1a, bhlhe41*, and *nr1d1*. Altered transcription of *per1, bhlhe41*, and *nr1d1* has been observed in the pituitary of sub-adult female Coho salmon exposed to EE_2_, and circadian rhythm genes were among the top affected pathways also in that study (Harding et al., 2013). In zebrafish, regulation of circadian rhythm genes in whole brain differs between fish with high- and low-stress sensitivity (Rey et al., 2013). Although circadian rhythm genes are not widely recognized as being estrogen responsive, several studies have found alterations in clock genes in response to estrogen exposure. In rodents, clock genes are altered in both the uterus and SCN in response to estrogen (Nakamura et al., 2001, 2005, 2008). Also, a connection has been found between the onset of locomotor activity and the different phases of the estrous cycle (see Krizo and Mintz, 2014 for review). Recent research in rodents shows that the circadian rhythm is highly responsive to gonadal steroids. Estrogens administered to ovariectomized rats have an effect on the period length and onset of locomotor activity (Krizo and Mintz, 2014). Photoperiod and melatonin, the major hormone in the circadian rhythm, is implied in the modulation of anxiety in rodents (Golombek et al., 1996; Nava and Carta, 2001; Trainor et al., 2007; Bilu and Kronfeld-Schor, 2013; Adamah-Biassi et al., 2014; Kumar et al., 2014; Nagy et al., 2014), possibly mediated by interaction with the GABAergic system (Golombek et al., 1996). Taken together, the observed effects on genes related to the circadian rhythm in female brain might represent a plausible connection to the stress sensitive behavior phenotype. No differential effects on *clock* genes were observed in male brains, but acute EE_2_ exposure has been previously found to upregulate hepatic *CRY2A* expression in male zebrafish (Wit et al., 2010) and downregulate *CLOCK* in fathead minnow testis (Garcia-Reyero et al., 2009). This study is, to the best of our knowledge, the first presenting RNA sequencing data in EE_2_ exposed male brains.

In males, the top putative pathway identified was cholesterol biosynthesis with four genes upregulated (Table 5). Cholesterol biosynthesis and metabolism has been found to be affected by estrogen in several previous studies (Cypriani et al., 1988; Hoffmann et al., 2006; Sharpe et al., 2007; Flores-Valverde et al., 2010; Hogan et al., 2010). Zebrafish embryos exposed to low concentrations of EE_2_ for 48 h (Schiller et al., 2013), as well as livers of adult zebrafish exposed to 30 ng/L EE_2_ for 4 or 28 days (Wit et al., 2010) showed up-regulation of genes involved in steroid biosynthesis and metabolism Also, steroidogenic enzymes were altered in fish brains by exposure to estrogenic compounds (Arukwe, 2005; Lyssimachou and Arukwe, 2007). This study identified up-regulated expression of four genes directly involved in cholesterol biosynthesis, *sqle, lss, msmo1*, and *hmgcs1*. RNAseq of brains of fluoxetine-exposed zebrafish males has recently revealed the same genes to be down-regulated, concomitant with anxiolytic effects in NT (Wong et al., 2013). Also, in a follow-up to the fluoxetine study, Wong et al. compared brain transcriptomes of two zebrafish lines specifically bred for different stress-coping behaviors (Wong et al., 2015). The results showed that fish with high stress sensitivity also had up-regulation of *sqle, lss, msmo1*, and *hmgcs1* compared to fish with lower stress sensitivity (Wong et al., 2015). In rodents, dietary cholesterol affects anxiety behavior (Hu et al., 2014), and reduced brain cholesterol was observed following chronic mild stress (Sun et al., 2015). Locally synthesized cholesterol is the precursor for neuro-steroids implied as potential mediators of anxiety in mammals (Gunn et al., 2011; Zorumski et al., 2013), augmenting GABA_A_ signaling (Gunn et al., 2011) but also interacting with NMDAR (Paul et al., 2013) and 5-HT1A receptor (Sun et al., 2015). If an association between cholesterol biosynthesis and anxiety in zebrafish males exist must await further confirmation, but it is intriguing to note a possible connection between GABA signaling and behavior in the top putative pathways in both males and females. Also, the association between cholesterol and neuro-steroids represent the only connection observed with the serotonergic system, via interaction with 5-HT1A.

In females, three genes with the ability to bind lipids were found to be down-regulated by developmental EE_2_ exposure (Table 4). One of these genes, *apoeb*, codes for the protein apolipoprotein Eb and is present in both brain and plasma. The human/mouse ortholog, apolipoprotein E, is produced largely by astrocytes and is believed to be one of the most important cholesterol transport proteins in the brain (Björkhem and Meaney, 2004). *Apoeb* mRNA levels has previously been found to be downregulated in adult zebrafish male, but upregulated in female, livers after exposure to 30 ng/L EE_2_ for 4 days (Wit et al., 2010). Downregulation of *abca12*, coding for an ABC-transporter, was also observed in the brains of EE_2_-exposed female zebrafish. Members of the ABC-transporter superfamily may be responsible for excluding circulating cholesterol from the brain (Björkhem and Meaney, 2004). However, the function of *abca12* remains largely unknown.

The current study identified six differentially expressed genes with recognized functions in the synapse. One of these genes, *sv2b*, encoding a synaptic vesicle protein (Bajjalieh et al., 1993) was upregulated in males developmentally treated with EE_2_ while *sv2b* was downregulated. Interestingly, two *sv2b* genes were also shown to be affected by fluoxetine treatment in male brains (Wong et al., 2013). In resemblance to the current study (Table 5), one *sv2b* paralogue was upregulated, while the other was down-regulated in exposed males (Wong et al., 2013). Also, cholesterol is essential in the process of myelination of axons (Björkhem and Meaney, 2004) and genes involved in cholesterol biosynthesis appear to play an important role in brain development and plasticity (Mathews et al., 2014). Among them is *hmgcs1*, one of the genes mentioned above to be upregulated by EE_2_ in male brains (Table 5). Knock-down of the encoded enzyme caused oligodendrocytes to migrate past their target axons (Mathews et al., 2014). Furthermore, the down-regulation of *arhgef9b* expression in male brains a gene, encoding collybistin, which has a pivotal role in the formation of postsynaptic glycine and inhibitory GABA receptor clusters (Kalscheuer et al., 2009), represent an additional change that might affect inhibitory GABA signaling (Kalscheuer et al., 2009). In females, an upregulation of brain *rtn4rl2a* expression, a gene whose product in humans and rodents is associated with prevention of axonal outgrowth (McDonald et al., 2011) and a regulator of synaptic plasticity (Lee et al., 2008), and of *bai1b* expression, coding for a negative regulator of brain angiogenesis that has recently been associated with synaptogenesis (Duman et al., 2013) was observed. In addition, *si:ch211-120g10.1*, a gene where the human ortholog *RNF39* encodes a protein suggested to play a part in synapse plasticity (Matsuo et al., 2001), showed decreased expression in exposed female brains. Taken together, EE_2_ exposure during zebrafish development induces several persistent alterations in the brain that likely affect the outcome of nerve signaling in the adult fish.

In the female brain, a surprisingly high number of differentially expressed novel miRNA was observed (Table 6). miRNAs cause modification of gene expression on posttranscriptional level, and are involved in sex differentiation in the brain (McCarthy and Nugent, 2015). Estrogens have been shown to regulate the expression and biogenesis of miRNA (Gupta et al., 2012; Klinge, 2012). It is possible that the differentially expressed miRNAs contributes to further effects at the protein level in the EE_2_-exposed female brain. As the function of the observed miRNAs are not known, no conclusions can be drawn and we can only report our findings awaiting future clarification of their function.

## Conclusions

This study has verified our previous findings of persistent effects on stress behavior in response to developmental exposure to low doses of EE_2_. RNA sequencing of male and female brain transcriptome revealed differences in differential expression between the sexes. No effects on genes belonging to the stress axis was observed in any of the sexes, but the expression of several genes in the regulation of circadian rhythm in females and cholesterol biosynthesis in males were found to be affected. Both pathways have previously been implied in anxiety regulation. Also, altered expression of several genes associated with synaptic function was observed, which might turn out to be important for the developmental modulations resulting in an anxiety phenotype. Further studies are needed to evaluate the significance of these findings, representing an initial survey of the effects of developmental exposure to the ubiquitous environmental contaminant EE_2_ on the brain transcriptome in the adult zebrafish.

## Author contributions

TP planned, designed, and conducted experiment, performed qPCR, prepared figures and tables and completed the manuscript. KV planned, designed, and conducted experiment, performed manual functional classifications of coding genes and prepared tables, and wrote first draft of the paper and thereafter reviewed drafts of the paper. NR planned, designed, and conducted experiment and reviewed drafts of the paper. TK performed bioinformatics and biostatistics and reviewed drafts of the paper. PD performed statistical analyses and prepared figures and reviewed drafts of the paper. IP participated in and held main responsibility for the project from planning of experiment to the final manuscript.

### Conflict of interest statement

The authors declare that the research was conducted in the absence of any commercial or financial relationships that could be construed as a potential conflict of interest.

## Supplementary material

Figure S1 **Experimental set up for behavioral tests seen from the side. (A)** Novel tank test. **(B)** Shoaling test. **(C)** Scototaxis test.

## Abbreviations

EDC: Endocrine disrupting compound
EE_2_: 17α-ethinyl estradiol
NT: novel tank
RNAseq: RNA sequencing
qPCR: quantitative Real Time polymerase chain reaction
ncRNA: non-coding RNA
miRNA: micro RNA.
