# Supplementary figures and images for: Persistent Effects of Developmental Exposure to 17α-Ethinylestradiol on the Zebrafish (Danio rerio) Brain Transcriptome and Behavior
Source: Front Behav Neurosci. 2017 Apr 20;11:69. doi: 10.3389/fnbeh.2017.00069 (PMC5397488; doi:10.3389/fnbeh.2017.00069)

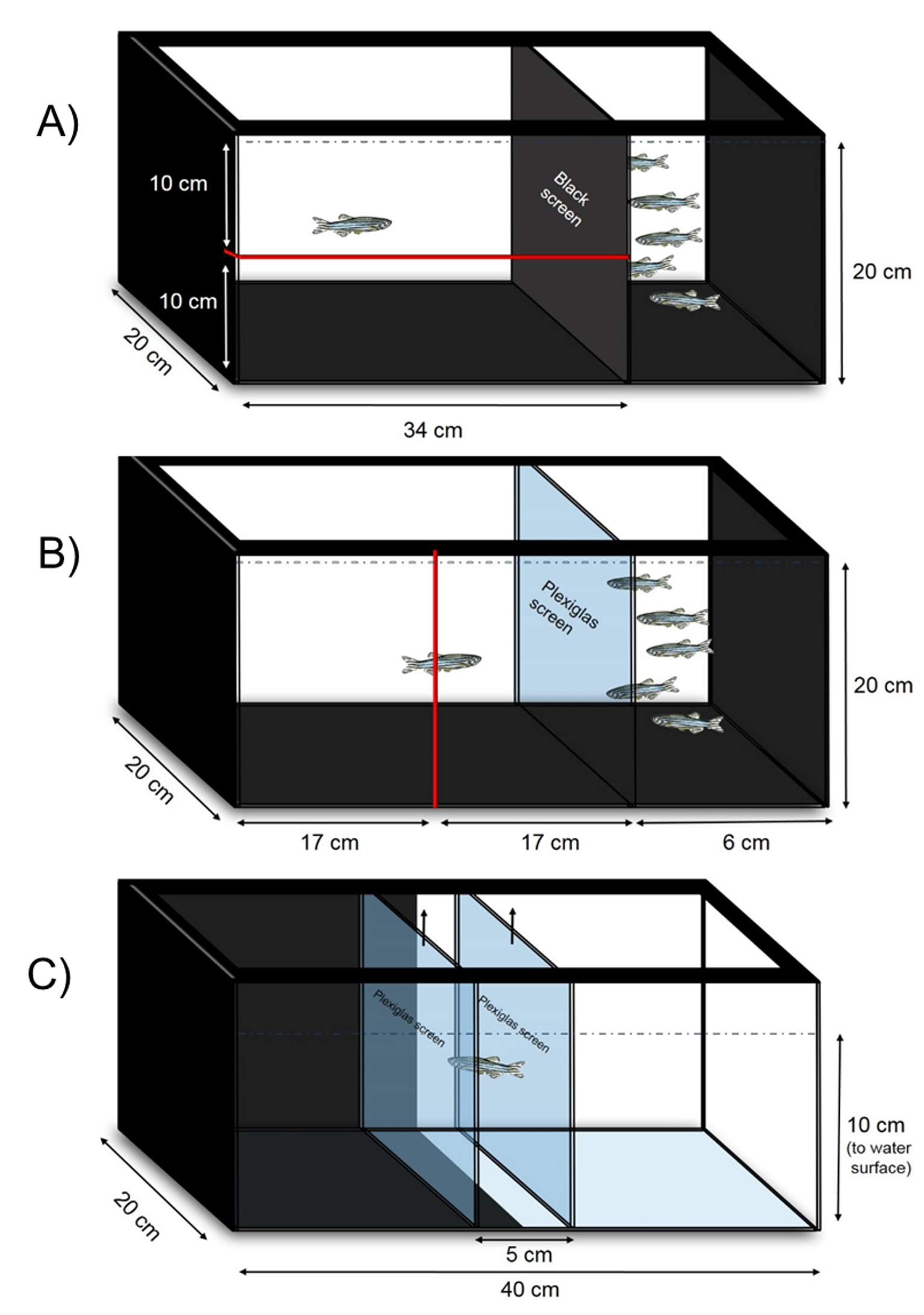

Supplement: Figure S1 — Experimental set up for behavioral tests seen from the side. (A) Novel tank test. (B) Shoaling test. (C) Scototaxis test. [file Image1.TIF]
